# Understanding the Epidemiology of Monkeypox Virus to Prevent Future Outbreaks

**DOI:** 10.3390/microorganisms12122576

**Published:** 2024-12-13

**Authors:** Jimmy Steven Velásquez, Fabiola Beatriz Herrera-Echeverría, Héctor Salvador Porres-Paredes, Carmen Rodríguez-Cerdeira

**Affiliations:** 1 Dermatology Department, Hospital Regional de Occidente San Juan de Dios, Quetzaltenango 09001, Guatemala; js.velasquezb@gmail.com (J.S.V.); salvaporres@yahoo.com (H.S.P.-P.); 2 Fundación Vithas, Grupo Hospitalario Vithas, 28043 Madrid, Spain; 3 Ibero-Latin American College of Dermatology (CILAD), Buenos Aires C1091, Argentina; 4 Dermatology Department, Hospital General San Juan de Dios, Guatemala 01001, Guatemala; fabiabeahe@gmail.com; 5 Dermatology Department, Grupo Hospitalario (CMQ Concheiro), Manuel Olivié 11, 36203 Vigo, Spain; 6 Department of Health Sciences, University of Vigo, Campus of Vigo, As Lagoas, 36310 Vigo, Spain

**Keywords:** monkeypox, MSM, HIV, genital ulcers

## Abstract

Monkeypox (Mpox) is an infectious disease caused by the Mpox virus belonging to the Orthopoxvirus genus in the Poxviridae family and has been declared by the WHO as a global health emergency owing to its rapid spread during 2022 and 2023. All patients diagnosed with Mpox who were confirmed by PCR between July 2022 and April 2023 were included in this study. In total, 405 patients in whom clade 2 was identified were included. Notably, 99% of included patients were men, with 82% of them aged 20–39 years. Furthermore, 71% were men who had sex with men, and 34% were HIV carriers. Regarding the morphology of the lesions, approximately 63% presented with papulonecrotic rash, which sometimes alternated with pustules depending on the stage they were in. All patients presented with systemic symptoms. Five patients required hospital admission, one of whom died, and presented with HIV and severe immunosuppression. Clinical findings suggest that contact during sexual intercourse is the most likely transmission mechanism and genital involvement is the most frequent clinical form. HIV was the primary comorbidity. Genital lesions were common, especially in vulnerable populations such as those who engage in high-risk sexual behaviors.

## 1. Introduction

In July 2022, the World Health Organization (WHO) declared monkeypox (Mpox) a public health emergency of international concern because of the unprecedented global spread of the disease outside of the previously endemic countries in Africa and the need for global solidarity to address this previously neglected disease. The 2022 outbreak has been primarily associated with close intimate contact (including sexual activity); most cases have been diagnosed among men who have sex with men and often present with novel epidemiological and clinical characteristics [1]. Patients present with a predominance of anogenital lesions, mucosal lesions, and other features, such as anorectal pain, proctitis, oropharyngeal lesions, tonsillitis, and multiphasic skin lesions. We have previously described the demographics and clinical spectra of classical and novel Mpox, outlining their potential complications and management [2].

Guatemala was one of the most affected countries in terms of the number of infected individuals [3].

The incubation period of Mpox ranges from 7 to 21 days. Criteria, such as little or no immunity at the population level and evidence of infection in all WHO regions, fit the definition of a pandemic [4].

In the existing literature, there are gaps in knowledge regarding the risk factors, sexual behaviors, and clinical manifestations in patients affected by this disease [5,6].

In this study, the aim was to describe the Mpox outbreak in Guatemala to obtain statistics to quickly recognize and address this entity. This is because timely diagnosis and treatment lead to decreased complications, mainly in groups with high-risk practices as well as in immunosuppressed patients [7,8]. Studying Mpox epidemiology is important for understanding specific host interactions, aids in ongoing outbreaks, and predicting future outbreaks.

## 2. Materials and Methods

This was a descriptive, cross-sectional, retrospective study of all patients diagnosed with Mpox. We confirmed Mpox virus infection through real-time PCR of samples from the mucosal and skin lesions of different parts of the patients’ bodies from July 2022 to April 2023 in the Epidemiology Department of Guatemala (6a Avenida 3-45 zona 11. Guatemala). This is a non-probabilistic and intentional sampling in which all patients who tested positive during the outbreak that occurred in Guatemala in 2022 were included. A total of 405 patients were included in this study.

The Dermatology Service of the San Juan de Dios General Hospital in Guatemala helped us complete the clinical and laboratory data of the patients studied there.

The variables studied included age, sex, sexual orientation, origin, clade, lesion morphology, location, probable transmission mechanism, immunization with smallpox vaccine, associated comorbidities, systemic impact, location in the genital area, and hospital admission.

### 2.1. Statistical Analysis

Descriptive analysis of the quantitative and qualitative variables was performed, and measures of central tendencies, percentages, and frequencies were used. Tables were used to combine the data and improve the analysis of variable. Statistical software SPSS Statistics 20 was use.

### 2.2. Ethical Aspects

This study was approved by the Teaching and Research Department of San Juan de Dios General Hospital. As this was a descriptive, cross-sectional, retrospective study, information was collected from the databases of the Epidemiology Department, taking into account the current regulations regarding data protection.

## 3. Results

Of the 405 patients included in this study, we found only one female patient (0.25%), with the male sex predominating with 99.75%. Most patients were aged 20–39 years (82%), with a peak between 25 and 29 years (29.14%). The mean age of the patients was 31 years (Table 1).

All patients were diagnosed with Mpox clade 2.

Table 2 shows the risk factors in patients diagnosed with Mpox by PCR in Guatemala; the distribution of the affected population according to sexual orientation is as follows: men who have sex with men 70.86%, bisexuals 17.28%. The population with the lowest number of cases is heterosexual, representing 8.64%. Furthermore, 81.96% of the population confirms previous sexual contact and 18.04% denies it. In relation to comorbidities, 33.82% present with HIV association as the main comorbidity, 2.73% with immunosuppression including chronic liver disease, cancer, and treatment with corticosteroids, each 0.49%, and a single case in a patient with chronic kidney disease representing 0.25%. Finally, only 1.98% had smallpox immunization; the rest did not have such immunization or did not know they had it. In immunized patients, the symptoms in general were attenuated and we did not find severe general repercussions.

Table 3 represents the clinical characteristics and morphology of cutaneous and extracutaneous lesions in patients diagnosed with Mpox. All patients presented with the following cutaneous manifestations: papulonecrotic/papulopustular lesions (62.96%), vesicular lesions (62.96%), macular lesions (54.07%), and in the crust phase (24.94%). Regarding the number of lesions, 82.96% presented <25, 19.79% with 25–50, and 0.25% with >50 cutaneous lesions.

**Figure 1:**
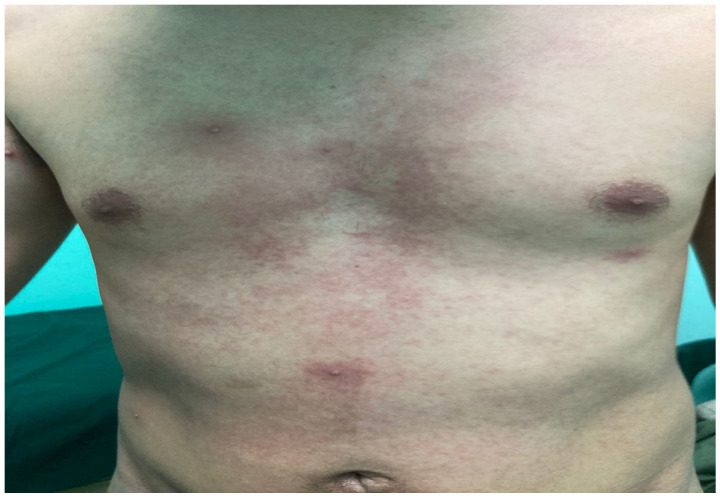
Rash and lesions at different stages in a man who has sex with men.

**Figure 2:**
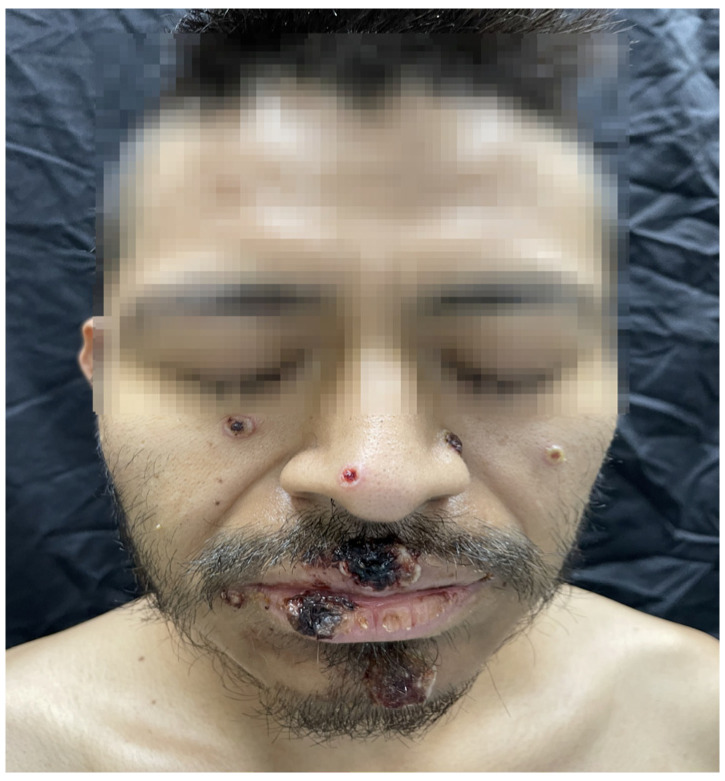
Multiple pseudopustules, some were crater-shaped with erosive centers.

**Figure 3:**
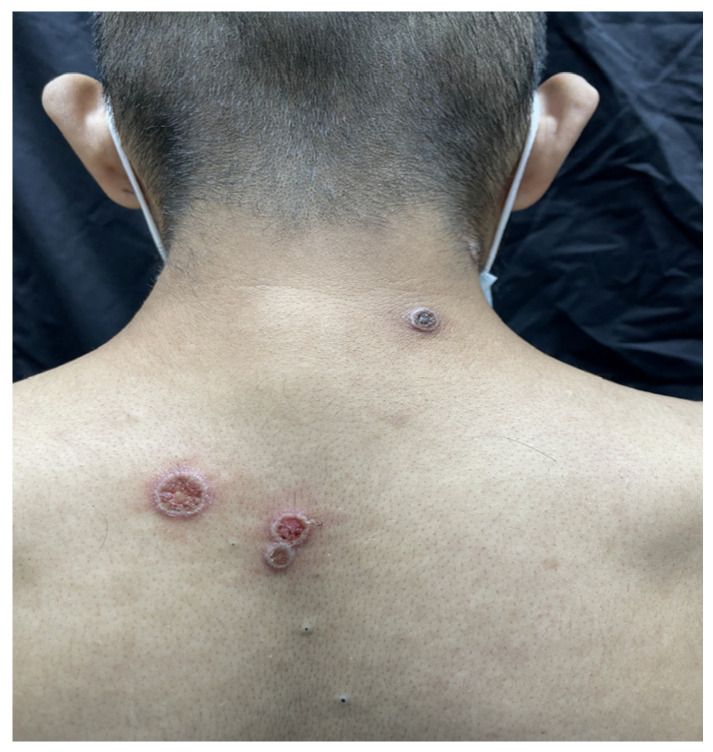
Regular circular ulcer with a white peripheral border, and purplish red central bottom.

The rash was localized in 28.89% of cases and disseminated in 71.11% of cases. The rash was present in different stages in 74.32% of cases and in the same stage in 25.68% of cases (Figure 1, Figure 2 and Figure 3).

Eleven patients with urethral discharge underwent a discharge swab for sexually transmitted diseases (STIs) panel screening; all panels were negative for the following microorganisms (*UU*, *Ureaplasma urealyticum*; *UP*, *Ureaplasma parvum*; *MG*, *mycoplasma genitalum*; *MH*, *mycoplasma hominis*; *NG*, *neisseria gonorrhoeae*; *CT*, *chlamydia trachomatis; vaginalis*). *Enterococcus faecalis* was isolated in the Gram stain and secretion culture of one of the patients, who also confirmed anal sexual relations, and all panel results are included in Table 4.

Five patients required hospital admission, one of whom died while also suffering from HIV and severe immunosuppression (Table 4).

**Figure 4:**
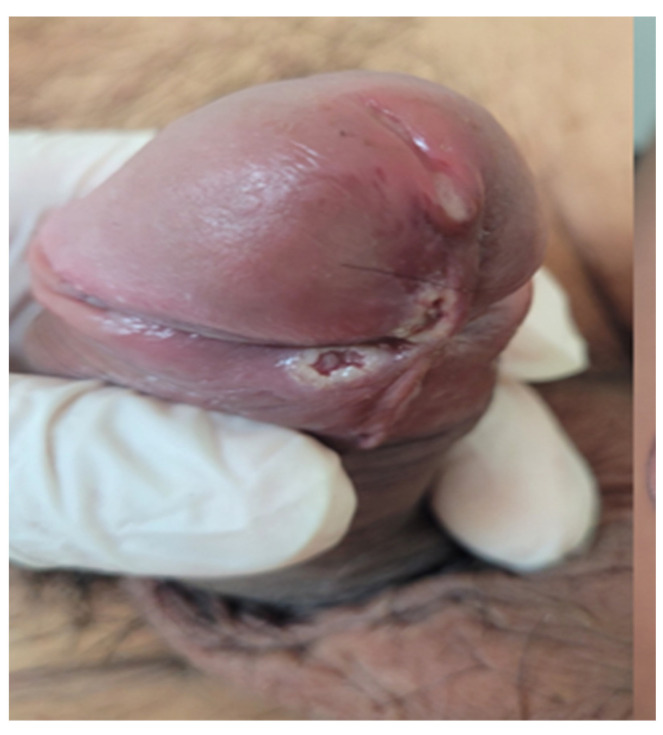
Lesion at inoculation site. Confluent ulcers with an associated depressed necrotic center.

**Figure 5:**
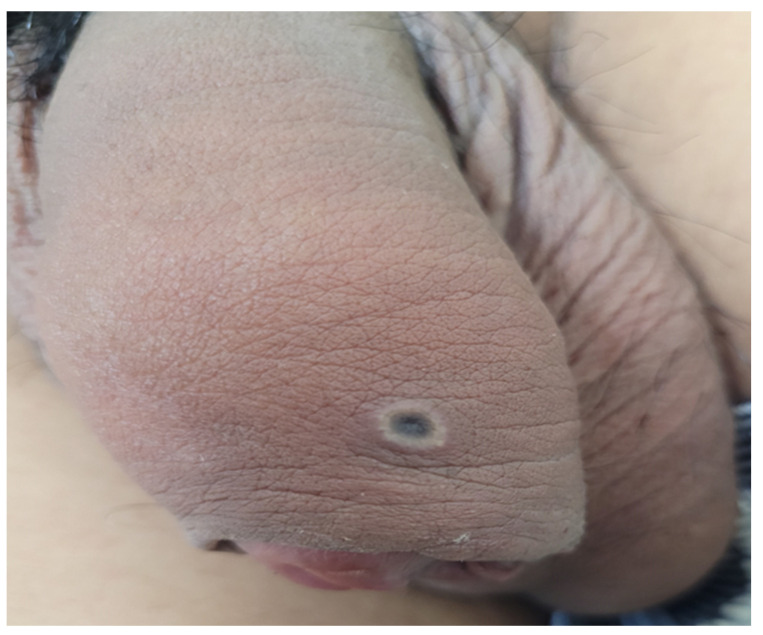
An umbilicated pustule on the dorsal surface of the penis.

All patients presented with genital involvement (Figure 4), which manifested as ulcerations (90.12%), papulonecrotic lesions (62.96%) (Figure 5), in the crusting phase (24–94%), and urethral discharge (1.98%). Genital lesions were asymptomatic in 97.04% and associated with pain or burning in 2.96%.

**Figure 6:**
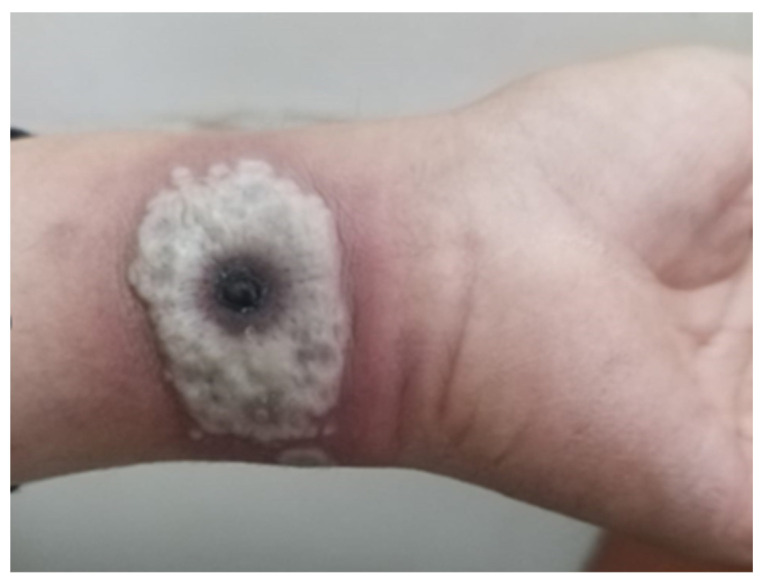
Papulonecrotic lesions and ulcerations with extensive necrosis in a patient with HIV.

**Figure 7:**
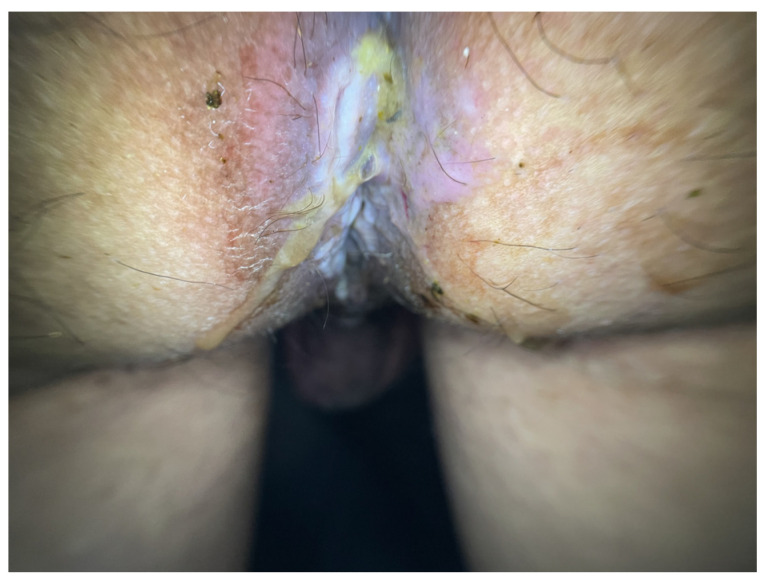
Several ulcerated lesions were observed on the penis in this patient VIH + and MSM.

The following extracutaneous manifestations were documented: fever (73.09%), headache (55.06%), myalgia (50.12%), regional lymphadenopathy (50.12%), and asthenia (35.06%). The rashes occurred after the extracutaneous manifestations in 47.9%, simultaneously in 46.91%, and before the extracutaneous manifestations in 5.19%. Notably, 1.23% of the patients required hospital admission, among whom one died due to advanced HIV infection and severe immunosuppression (Figure 6 and Figure 7).

Our patients received only symptomatic treatment, depending on the pathology they presented and the associated comorbidities. There are no updated and approved antiviral treatments available at this time.

## 4. Discussion

Clade 2 was identified in all the patients included in this study. Clade 1 (with case fatality rates of 1–12%) is usually responsible for the disease in Central Africa and the Congo Basin, whereas clade 2 (which is less virulent, with case fatality rates of <0.1%) is found in West Africa. Molecular genome analysis performed in Spain indicated that the Mpox detected in Spain was a part of the West African clade and was a close analogue to other genomes identified in other European countries and Latin America [9,10,11].

Regarding sexual orientation, the distribution was as follows: 40.74% were homosexual individuals, 30.12% were MSM, and 17.28% were bisexual individuals. Combining the above data, the majority of the study population was composed of MSM individuals (88.11%), and the population with the lowest number of cases was heterosexuals (8.64%). Furthermore, 81.96% of the study population confirmed previous sexual contact, whereas 18.04% denied it. Similar to other epidemiological studies, the outbreaks occurred more frequently among men and young individuals [12,13].

The largest proportion of male patients belonged to MSM individuals [14,15].

Skin lesions usually appear primarily in the genital area and then spread to other areas [16].

Once disseminated, the morphological characteristics are in the form of a pseudopustule with a necrotic-hemorrhagic center, although sometimes the characteristics are completely atypical and clinical variations have been recorded with respect to the clinical picture previously described in endemic areas [17,18].

We detected Mpox viral DNA in both skin lesions and seminal fluid [19,20].

The same is true for rectal swab samples [19,20]. Furthermore, Mpox viral DNA has been detected in respiratory secretions [21] and the blood of patients during the 2022 outbreak [21,22].

Lapa et al. demonstrated the persistence of Mpox DNA in the semen of infected individuals for 19 days [20].

Peiro-Mestres et al. found that the viral loads in skin lesion samples were of the same magnitude as those in semen samples [19].

Moschese et al. cultured Mpox virus from urethral swabs in 11 of 15 PCR-positive cases and from 13 rectal swabs in 18 PCR-positive cases [23].

This demonstrates that sexual transmission of Mpox is biologically possible.

Individuals aged >50 years may experience more severe Mpox disease owing to a higher prevalence of comorbidities. Conversely, they may have residual protection against severe Mpox infection from childhood smallpox vaccination, as suggested by an investigation of previous Mpox outbreaks [24].

Mpox may affect multiple organ systems; therefore, individuals with comorbidities, including HIV and immunocompromised conditions, may be at an increased risk of more severe Mpox disease [25,26].

The disease appears to have a lethal prognosis, especially in children who have not been vaccinated against smallpox [27,28].

Differential diagnoses include chickenpox, herpes zoster infection, measles, Zika virus infection, dengue, chikungunya, herpes simplex, impetigo, methicillin-resistant *Staphylococcus aureus* infection, disseminated or localized gonorrhea, primary or secondary syphilis, chancroid, lymphogranuloma venereum, and molluscum contagiosum.

Currently there are no specific treatments for Mpox, and symptomatic treatment is the therapeutic pillar.

It is known that the vaccinia vaccine can offer partial protection against Mpox infection. However, the use of smallpox vaccines for Mpox prevention in epidemic regions is limited due to the potential risks for immunocompromised individuals, particularly those co-infected with HIV [29].

Finally, so far, only a few antiviral compounds have been approved by the regulatory authorities. Given the high mutability of the Mpox virus, certain mutant strains have shown resistance to existing pharmaceutical interventions [30].

Furthermore, we have gained valuable insights from the process of Mpox drug development, including strategies for repurposing drugs, the discovery of drug targets driven by artificial intelligence, and preclinical drug development. The development of antiviral drugs and vaccines against monkeypox virus is urgently needed, despite some therapeutic effects of currently used drugs in the clinic [31].

## 5. Conclusions

The transmission dynamics of Mpox during the current outbreak were highly consistent with sexually transmitted infections. Therefore, a sexual health framework must be incorporated into the outbreak response.

Targeted screening of populations at high risk of other sexually transmitted infections may be an important strategy for case identification.

Recent experimental studies and careful epidemiological analyses have demonstrated the transmissibility of Mpox through different body fluids.

Anogenital lesions often occur before disease dissemination, suggesting direct inoculation during sexual activities.

Additionally, attention should be paid to the possibility that different transmission dynamics exist across different regions of the world.

We advocate the importance of public health interventions such as vaccinations, complementary testing, and early treatment, as well as the provision of awareness and education programs focused on behavioral modifications to reduce exposure.

Finally, studying Mpox epidemiology is important to understand specific host interactions, aid in ongoing outbreaks, and predict future outbreaks.

## Figures and Tables

**Table 1:** Epidemiological characteristics of the patients.

Sex		%
Male	404	(99.75)
Female	1	(0.25)
Age (years)		%
60–64	1	(0.25)
55–59	2	(0.49)
50–54	6	(1.48)
45–49	15	(3.70)
40–44	39	(9.63)
35–39	57	(14.07)
30–34	98	(24.20)
25–29	118	(29.14)
20–24	58	(14.32)
15–19	10	(2.47)
10–14	1	(0.25)
Clade		%
Clade 1	0	(0.00)
Clade 2	405	(100)

**Table 2:** Risk factors and comorbidities.

Sexual Orientation		%
Men who have sex with men (MSM)	287	(70.86)
Bisexual	70	(17.28)
Heterosexual	35	(8.64)
Unspecified	13	(3.22)
Previous sexual contact?		%
Yes	332	(81.96)
No	73	(18.04)
Travel history	22	(30.14) (22/73)
Wild animal contact	12	(16.44) (12/73)
Unspecified	39	(53.42) (39/73)
Smallpox immunization *		%
Immunization history	8	(1.98)
Immunized denied	397	(09.02)
Comorbidities	%	
None	250	(61.73)
HIV	137	(33.82)
Immunosuppression (non-specified)	11	(2.73)
Chronic liver disease	2	(0.49)
Cancer	2	(0.49)
Treatment with corticosteroids	2	(0.49)
Chronic kidney disease	1	(0.25)
Smallpox immunization *		%
Immunization history	8	(1.98)
Immunized denied	397	(09.02)
Comorbidities (HIV)	6	1.48%
Condition severity (mild)	8	1.98%

* Number of patients immunized.

**Table 3:** Clinical characteristics.

Elementary Skin Lesion		%
Papulonecrotic	255	(62.96)
Vesicular	255	(62.96)
Macular	219	(54.07)
Crust	101	(24.94)
Number of skin lesions		%
<25 lesions	336	(82.96)
25–50 lesions	68	(19.79)
>50 lesions	1	(0.25)
Location of the rash		%
Located	117	(28.89)
Disseminated	288	(71.11)
Morphology of the rash		%
Polymorphic dermatosis (exanthema in different stages)	301	(74.32)
Monomorphic dermatosis (exanthema at the same stage)	104	(25.68)
Extracutaneous manifestations		%
Fever	296	(73.09)
Headache	223	(55.06)
Myalgia	203	(50.12)
Regional lymphadenopathy	203	(50.12)
Asthenia	142	(35.06)
Evolution of the rash		%
After extracutaneous manifestations	194	(47.90)
Simultaneous	190	(46.91)
Previous extracutaneous manifestations	21	(5.19)
Genital lesions		%
Ulcerations	365	(90.12)
Papulonecrotic	255	(62.96)
Crusts	101	(24.94)
Urethral discharge	8	(1.98)
Symptoms associated with genital lesions		%
Painless	393	(97.04)
Pain/burning	12	(2.96)

**Table 4:** Laboratory test and complications.

		Total Patients (405)
Type of swab performed		%
Exudate swab	279	(68.89)
Lesion surface swab	375	(92.59)
Scab swab	258	(63.70)
STI PCR		%
STI PCR performed STI PCR negative	11 11	(2.72) (100) (11/11)
STI PCR not performed	394	(87.28)
Gram stain and bacterial culture		%
Performed Fibrin *Enterococcus faecalis*	11 11 1	(2.72) (100) (11/11) (9.09) (1/11)
Not performed	394	(87.28)
Syphilis test (FTA-ABS)		%
Performed Antibodies detected	137 18	(33.83) (13.13) (18/137)
Not performed	268	(66.17)
Complications		%
Hospitalization	5	(1.23)
Superinfection	5	(1.23)
Pneumonia	1	(0.25)
Death	1	(0.25)

## Data Availability

The original contributions presented in the study are included in the article, further inquiries can be directed to the corresponding author.
